# A Decreasing North-to-South Gradient of *HFE* p.C282Y (rs1800562) Allele Frequencies in Iberia: An Analysis of 34 Population/Control Cohorts

**DOI:** 10.3390/genes17030277

**Published:** 2026-02-27

**Authors:** James C. Barton, J. Clayborn Barton, Ronald T. Acton

**Affiliations:** 1 Southern Iron Disorders Center, Birmingham, AL 35209, USA; jbarton205@gmail.com (J.C.B.); rtakma@bellsouth.net (R.T.A.); 2 Department of Medicine, The University of Alabama at Birmingham, Birmingham, AL 35294, USA; 3 Department of Microbiology, The University of Alabama at Birmingham, Birmingham, AL 35294, USA

**Keywords:** cline, hemochromatosis, *HFE* p.C282Y heterozygote, latitude, longitude, Portugal, Spain

## Abstract

**Background:** We sought to analyze the geographic distribution of *HFE* p.C282Y (homeostatic iron regulator c.845G>A; rs1800562) allele frequencies in Iberia. **Methods:** We analyzed published population/control cohorts of 50 or more subjects in mainland Spain and mainland Portugal and determined whether or not the p.C282Y genotypes in each cohort deviated from Hardy-Weinberg equilibrium (HWE) proportions. We defined combined p.C282Y allele frequencies from Spain and Portugal as representative of Iberia. We computed linear regressions (Pearson’s correlations) of allele frequencies vs. latitudes and longitudes of cohort recruitment sites, defined significant regressions as allele frequency gradients, and mapped regional allele frequencies. **Results:** There were 34 Iberian cohorts: 25 Spanish (12,297 subjects; 11 autonomous communities) and 9 Portuguese (1024 subjects; five administrative regions). p.C282Y genotypes in one of 34 cohorts (2.9%) deviated significantly from HWE proportions. Aggregate allele frequency in Iberia was 0.0292 (778/26,642) [95% confidence interval: 0.0272, 0.0313]. The correlation of allele frequencies with latitude in Iberia was significant (r_34_ = 0.4184; *p* = 0.0138). The correlation of allele frequencies with longitude was not significant (r_34_ = 0.0014; *p* = 0.9936). The range of 16 regional allele frequencies in Iberia was 0.0068 (Murcia) to 0.5000 (Galicia). Frequencies were highest in regions adjacent to the north and northwest coasts (Cantabria, Galicia, Norte) and lowest in the south (Algarve, Murcia). **Conclusions:** There is a significant decreasing linear north-to-south gradient of *HFE* p.C282Y allele frequencies in Iberia. p.C282Y allele frequencies are highest in regions adjacent to the north and northwest coasts.

## 1. Introduction

*HFE*, the homeostatic iron regulator (chromosome 6p22.2) [1,2], encodes the non-classical class I major histocompatibility complex protein HFE, an upstream modulator of the central iron-regulatory hormone hepcidin (*HAMP,* chromosome 19q13.12) [3]. *HFE* p.C282Y (c.845G>A; rs1800562) is a common missense mutation in persons of European ancestry that often occurs in linkage disequilibrium with the human leukocyte antigen (HLA) locus A*03, the marker of the ancestral p.C282Y haplotype [4,5,6]. It has been estimated that *HFE* p.C282Y arose before 4000 BCE [7,8]. The discovery of p.C282Y heterozygosity in a Bronze Age man from a cist burial (2026-1885 BCE) on Rathlin Island, County Antrim, Ireland [9] is consistent with this estimate. p.C282Y homozygosity is associated with the predominant subtype of hemochromatosis [2,10].

Iberia, also known as the Iberian Peninsula, is a landmass of 583,544 km^2^ (225,308 square miles) in southwestern Europe which is separated from the rest of Europe by the Pyrenees Mountains [11]. The area of Iberia is predominantly that of mainland Spain (84.5%) and mainland Portugal (15.3%) [11]. The remaining area comprises the microstate Andorra and a small part of the French department of Pyrénées-Orientales in the northeast, and in the south, Gibraltar, a British Overseas Territory [11].

*HFE* p.C282Y allele frequencies in Iberia are lower than those of most northwestern European countries, although few data support this observation [12,13,14]. In a study of four cohorts, Mónzo et al. [15] proposed that there is a decreasing west-to-east gradient of p.C282Y allele frequencies in Spain. Cardoso et al. demonstrated that p.C282Y allele frequencies were significantly higher in the north and central regions of Portugal than in the south [16].

The goal of this study was to evaluate the relationships of *HFE* p.C282Y allele frequencies with latitudes and longitudes in Iberia using data from 34 published population/control cohorts (13,321 subjects, 16 geographic regions) [17]. We determined whether or not the numbers of subjects with p.C282Y genotypes in each population/control cohort deviated from Hardy-Weinberg equilibrium (HWE) proportions. We computed linear regressions (Pearson’s correlations) of p.C282Y allele frequencies vs. latitudes and longitudes of cohort recruitment sites, defined significant regressions as frequency gradients, and mapped the p.C282Y allele frequencies of the geographic regions. We discuss possible causes of variability in the present p.C282Y allele frequencies and explore the putative selective advantages of p.C282Y heterozygosity and evidence of migrations pertinent to p.C282Y in Iberia.

## 2. Methods

### 2.1. Definition of Population/Control Cohort

We defined a population cohort as a group of research subjects who share a common characteristic(s) and are used in a study to represent the broader population. We defined a control cohort as a group of individuals in a study who do not have the condition or outcome of interest but who are otherwise similar to the individuals in the main study group and are presumed to represent the broader population. In this study, we defined population and control cohorts to be equivalent.

### 2.2. Definition of Evaluable Population/Control Cohorts

We defined evaluable cohorts as those in which the corresponding reports included all of the following data: (1) 50 or more population/control subjects [18]; (2) the attributes of the population/control subjects; (3) the nominal geographic site of subject recruitment (or location of the primary investigator’s institution, as available); and (4) determinable numbers of *HFE* p.C282Y and total alleles and p.C282Y genotypes.

### 2.3. Evaluable Population/Control Cohorts Included

We tabulated *HFE* p.C282Y allele frequencies in 25 population/control cohorts (12,297 subjects) from 11 autonomous communities of mainland Spain (Aragon, Asturias, Basque Country, Cantabria, Castile-La Mancha, Catalonia, Extremadura, Galicia, Madrid, Murcia, and Valencia) and nine population/control cohorts (1024 subjects) from the five administrative regions of Portugal (Alentejo, Algarve, Centro, Lisbon-Tagus Valley, and Norte) as described in detail elsewhere [17]. We tabulated the latitudes and longitudes [17,19] of the nominal sites of cohort recruitment (or locations of primary investigator institutions, as appropriate) [17].

### 2.4. Evaluable Population/Control Cohorts Not Discovered

We did not discover evaluable cohorts from north-central Spain (Castile and León, La Rioja, and Navarre), south-central Spain (Andalusia), Andorra, Pyrénées-Orientales, or Gibraltar [17].

### 2.5. Population/Control Cohorts Excluded

We did not tabulate data from reports that described the following: (1) Roma people and other residents of Iberia who were not regarded as Iberian natives by the corresponding investigators; (2) no geographic region of cohort recruitment other than country; (3) *HFE* p.C282Y allele frequencies estimated using population prevalences of p.C282Y homozygotes; and (4) the publication of a previously reported population/control cohort. We excluded cohorts from the Balearic and Canary Islands (Spain), the Azores and Madeira (Portugal), and the autonomous cities of Ceuta and Melilla in Northern Africa (Spain) because these geographic regions are not in Iberia [11].

### 2.6. Statistics

All data analyzed in this study are presented herein or are openly available online [17]. These population/control cohorts were published during the period of 1997–2012 [17].

We determined whether or not the numbers of subjects with *HFE* p.C282Y genotypes (p.C282Y homozygosity, p.C282Y heterozygosity, and no p.C282Y) in each population/control cohort deviated from HWE proportions. Because 33 of the 34 cohorts (97.1%) had fewer than five subjects with p.C282Y homozygosity [20], we used HWE exact tests [21] with mid-p adjustments [22].

We computed the *HFE* p.C282Y allele frequency for each cohort as the quotient of (number of p.C282Y alleles) by (number of subjects × 2), expressed to four decimal places [95% confidence interval]. We defined combined p.C282Y allele frequency data from mainland Spain and mainland Portugal as representative of Iberia [17]. We used Fisher’s exact test (two-tailed) or the Chi-square test (two-tailed) to compare proportions, as appropriate.

In preliminary data exploration, we observed that there were sufficient cohorts from Catalonia/Barcelona (five cohorts; 7159 subjects, including 1043 randomly selected newborn screening cards) and Madrid/Madrid (five cohorts; 1925 subjects, including 1000 neonates) to evaluate for possible effects of the following variables on *HFE* p.C282Y allele frequencies: (1) geographic region; (2) cohort sample size; and (3) year of cohort recruitment (or year of publication). There were insufficient mean/median age data in the corresponding reports to evaluate age as a variable. We expressed p.C282Y allele frequencies as proportions in Chi-square tests (two-tailed) and as decimals in Pearsons correlation analyses.

*HFE* p.C282Y allele frequencies did not differ significantly in the five Catalonia/Barcelona population/control cohorts (Chi-square = 6.0540; *p* = 0.1952) or the five Madrid/Madrid population/control cohorts (Chi-square = 4.7336; *p* = 0.3157). Pearson’s correlations of cohort sample sizes vs. p.C282Y allele frequencies were not significant in the five Catalonia/Barcelona cohorts (r_5_ = −0.6001; *p* = 0.1423) or the five Madrid/Madrid cohorts (r_5_ = −0.0018; *p* = 0.4988). Pearson’s correlations of year of cohort recruitment (or year of publication) vs. p.C282Y allele frequencies were not significant in the five Catalonia/Barcelona cohorts (r_5_ = −0.7154; *p* = 0.1743) or the five Madrid/Madrid cohorts (r_5_ = −0.0769; *p* = 0.9022).

Kolmogorov-Smirnov testing indicated that the distribution of *HFE* p.C282Y allele frequencies we tabulated [17] did not differ significantly from those that are normally distributed. Thus, we computed linear regressions (Pearson’s correlations) of p.C282Y allele frequencies vs. latitudes and longitudes in decimal degrees (four decimal places) [19] and defined significant regressions as gradients. We defined the strengths of significant Pearson’s correlations according to these ranges of correlation coefficients (r): ≥ 0.80, very strong; 0.60–0.80, moderately strong; 0.30–0.59, fair; and < 0.30, poor [23]. We used the equations derived from significant linear regressions to estimate the changes in p.C282Y allele frequencies over distance. We defined 1.0000 degrees of latitude as 111.1 km (69.0 miles) [24].

We mapped *HFE* p.C282Y allele frequencies for 16 geographic regions (11 autonomous communities of mainland Spain and 5 administrative districts of mainland Portugal) of Iberia [17] using MapChart 2025 [25]. We used single values of p.C282Y frequency as regional frequencies, as necessary. For two or more p.C282Y frequency reports from the same geographic region [17], we computed the regional p.C282Y allele frequency as the quotient of (total number of p.C282Y alleles) by (total number of subjects × 2).

We used Excel^®^ 2000 (Microsoft Corp., Redmond, WA, USA) and GraphPad Prism 8^®^ (2018; GraphPad Software, San Diego, CA, USA). We defined values of *p* < 0.05 to be significant.

## 3. Results

### 3.1. Characteristics of 34 Population/Control Cohorts

We identified 25 cohorts in mainland Spain (12,297 subjects; 11 of the 15 autonomous communities) (Table 1) and nine cohorts in mainland Portugal (1024 subjects; each of the five administrative regions) (Table 2). The attributes of each cohort are described in detail elsewhere [17]. Cohorts were recruited in this region: latitude 43.4619–37.2299° N; longitude −9.1366–2.1899° W [17]. The latitude and longitude we analyzed for each cohort are displayed in detail elsewhere [17]. The range of p.C282Y allele frequencies in the 34 cohorts was 0.0000 to 0.0517 [17] (Table 1 and Table 2).

### 3.2. Hardy–Weinberg Equilibrium Proportions

In the 25 population/control cohorts from mainland Spain, numbers of *HFE* p.C282Y genotypes in one cohort (4.0%) deviated significantly from HWE proportions (Table 1). In the nine population/control cohorts from mainland Portugal, none deviated significantly from HWE proportions (Table 2). The ratios of cohorts in mainland Spain and mainland Portugal that deviated from HWE proportions did not differ significantly (1/25 vs. 0/9, respectively; *p* = 0.7353). Together, p.C282Y genotypes in 2.9% (1/34) of the present Iberian population/control cohorts deviated significantly from HWE proportions.

### 3.3. Aggregate HFE p.C282Y Allele Frequencies

The aggregate p.C282Y allele frequency in mainland Spain was 0.0291 (716/24,594) [0.0271, 0.0313] (Table 1). The aggregate p.C282Y allele frequency in mainland Portugal was 0.0303 (62/2048) [0.0237, 0.0386] (Table 2). These frequencies did not differ significantly (*p* = 0.8170). The aggregate p.C282Y allele frequency in Iberia was 0.0292 (778/26,642) [0.0272, 0.0313].

### 3.4. HFE p.C282Y Allele Frequencies vs. Latitudes and Longitudes in Spain

**Figure A1:**
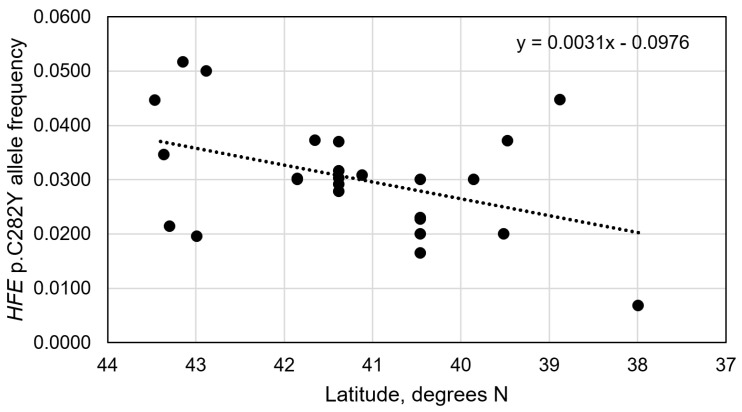
Pearson’s correlation of *HFE* p.C282Y allele frequencies vs. latitude in Spain (r_25_ = 0.4287, R^2^ = 0.1838; *p* = 0.0325).

The correlation of allele frequencies with latitude in the 25 population/control cohorts in mainland Spain was significant (r_25_ = 0.4287, R^2^ = 0.1838; *p* = 0.0325) (Figure A1). The strength of this correlation was fair. The regression line represents a 1.8-fold decrease in estimated allele frequency from 0.0371 in the north to 0.0202 in the south over a distance of 607.7 km (377.6 miles) (Figure A1). The correlation of allele frequencies with longitude was not significant (r_25_ = −0.1212, R^2^ = 0.0144; *p* = 0.5672).

### 3.5. HFE p.C282Y Allele Frequencies vs. Latitudes and Longitudes in Portugal

The correlation of allele frequencies with latitude in the nine population/control cohorts in mainland Portugal was not significant (r_9_ = 0.4316, R^2^ = 0.1863; *p* = 0.2461). The correlation of allele frequencies with longitude was not significant (r_9_ = −0.0196, R^2^ = 0.0004; *p* = 0.9796).

### 3.6. HFE p.C282Y Allele Frequencies vs. Latitudes and Longitudes in Iberia

**Figure 1:**
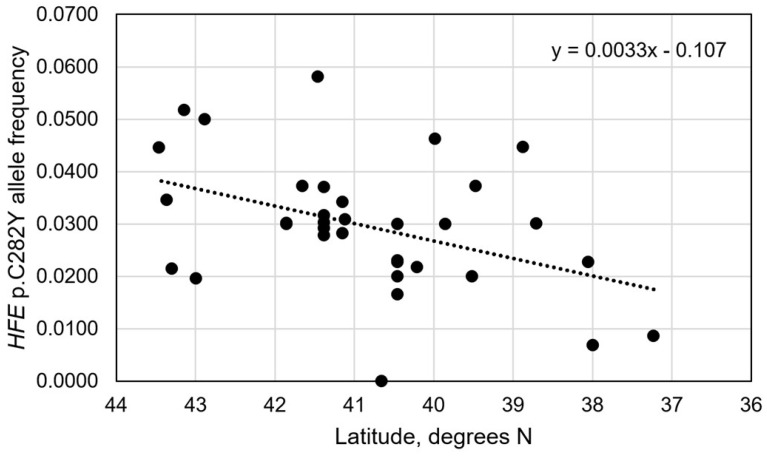
Pearson’s correlation of *HFE* p.C282Y allele frequencies vs. latitude in Iberia (r_34_ = 0.4184, R^2^ = 0.1751; *p* = 0.0138).

The correlation of allele frequencies with latitude from the 34 population/control cohorts in Iberia was significant (r_34_ = 0.4184, R^2^ = 0.1751; *p* = 0.0138) (Figure 1). The strength of this correlation was fair. The regression line represents an 2.3-fold decrease in estimated p.C282Y allele frequency from 0.0371 in the north to 0.0159 in the south over a distance of 692.4 km (430.2 miles) (Figure 1). The correlation of allele frequencies with longitude was not significant (r_34_ = 0.0014, R^2^ < 0.0001; *p* = 0.9936).

### 3.7. Regional HFE p.C282Y Allele Frequency Map of Iberia

**Figure 2:**
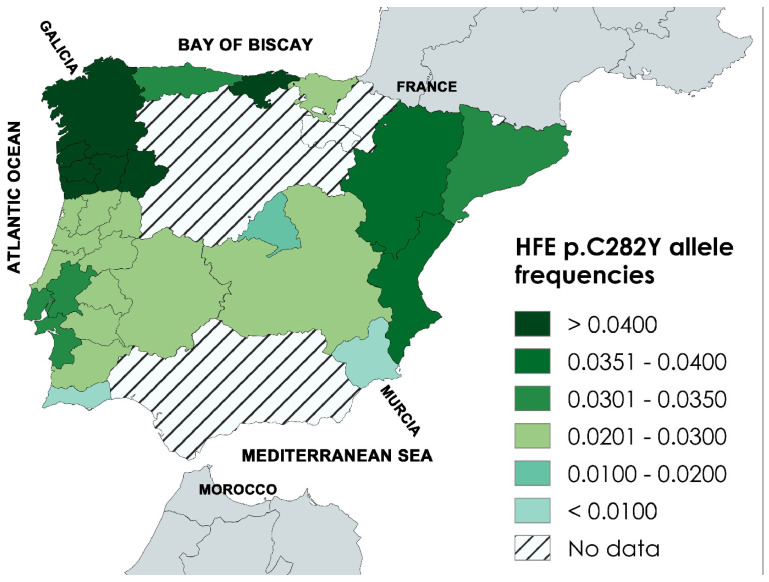
Regional *HFE* p.C282Y allele frequencies in 16 regions of Iberia (11 autonomous communities of Spain and five administrative regions of Portugal).

*HFE* p.C282Y allele frequencies, available from 16 regions of Iberia, differed 7.4-fold [17] (Table 3). The highest regional allele frequencies were observed in the north and northwest (Cantabria, Galicia, and Norte) (Table 3) (Figure 2). The lowest regional allele frequencies were observed in the southwest (Algarve) and the southeast (Murcia) (Table 3) (Figure 2).

## 4. Discussion

A novel finding of this study of 34 population/control cohorts is that there is a significant linear gradient of *HFE* p.C282Y allele frequencies that decreases from north to south in Iberia. This extends findings of other studies that similarly demonstrated that there are significant linear gradients of p.C282Y allele frequency that decrease from north to south across multiple European countries [13,52].

A second novel finding of this study is that there is no west-to-east linear gradient of *HFE* p.C282Y allele frequency in mainland Spain, mainland Portugal, or Iberia, in contrast to the proposal of Monzó et al. in their study of four Spanish cohorts [15]. Guix et al. demonstrated that there is a significant west-to-east linear gradient of p.C282Y allele frequency across 14 European national cohorts [13].

A third novel finding of this study is that the highest *HFE* p.C282Y allele frequencies in Iberia occur in the regions adjacent to the north and northwest coasts. In Denmark, Norway, Sweden, the Faroe Islands, Iceland, and eastern England and Ireland, the highest p.C282Y allele frequencies have been observed among populations living along the coastlines [14].

The *HFE* p.C282Y genotypes in only one of the present 34 Iberian population/control cohorts deviated significantly from HWE proportions. Natural selection, gene flow (migration), genetic drift (random change in p.C282Y frequencies in a region due to small populations, bottlenecks, and founder effects), mutation, and non-random mating could account for this HWE deviation [53]. Non-random selection of study subjects (sampling bias) [54] and genotyping errors [55,56] can also cause significant deviation from HWE proportions. Other factors that may have contributed to the variability of the present p.C282Y frequencies include differences in criteria for selecting population/control cohorts [57,58]; inadequate numbers of population/control subjects per cohort, especially in cohorts recruited from regions with low p.C282Y frequencies [59,60]; and cultural factors [61].

Do *HFE* p.C282Y heterozygotes have a selective advantage? In 1979, Motulsky postulated that heterozygotes for the hemochromatosis gene, especially women, have lower risks of iron deficiency [62]. In contrast, adults with p.C282Y heterozygosity did not absorb more heme and non-heme iron than adults with *HFE* wt/wt(absence of both p.C282Y and *HFE* p.H63D (rs1799945)) [63,64]. In 23,681 Caucasian adults, the prevalence of iron-deficiency anemia did not differ significantly between adults with p.C282Y heterozygosity and those with wt/wt [65]. In 62,685 women, p.C282Y prevalence did not differ significantly between those with or without iron deficiency, regardless of race/ethnicity, age subgroup, or pregnancy [66].

Other favorable traits attributed to *HFE* p.C282Y heterozygosity include the following: adaptation to Neolithic iron-poor diets [67,68]; mitigation of celiac disease [69]; adaptation to culture and climate [70]; increased resistance to infectious bacteria [71] or parasites [72]; superior physical performance [73]; higher hemoglobin levels [65]; altitude-induced compensatory erythrocytosis [74]; reproductive advantage [75]; and greater life expectancy [76]. It is uncertain whether or not heterozygosity for p.C282Y alone was advantageous for ancient (or present-day) Iberians. “Finally, one should not forget that the *HFE* gene is imbedded in the immune response region of the genome on chromosome 6. Perhaps the mutation is just a hitchhiker, being carried along with a group of immune response genes that are favorable for survival” [77].

Simon et al. hypothesized that *HFE* p.C282Y arose in Celtic people in central Europe and was spread by their migrations [78], an explanation supported by further analyses of historical events, geography, and p.C282Y frequencies [14,52,79]. Archaeologic and linguistic evidence suggests that Celts from central Europe gradually occupied Iberia over many centuries before the Christian era [80]. During the Late Bronze Age (c. 1300 BCE–c. 600 BCE), Celts in northwest Iberia maintained social and commercial relations with people who lived in present-day Brittany, the Cornish Peninsula, Wales, and Ireland [80]. Archaeologic, historic, and genetic evidence suggests that Celts were the dominant populations in the Iberian Peninsula by c. 500 BCE [14].

*HFE* p.C282Y allele frequencies are relatively high in areas of Europe with large present-day populations of Celtic descent, including Ireland, Scotland, England, and Brittany [81]. The highest known p.C282Y allele frequencies occur in areas adjacent to the east coast of Ireland (Dublin 0.1422, Belfast 0.0990) [82,83]. In the present study, p.C282Y allele frequencies were highest in Cantabria, Galicia, and Norte, all regions in the “Celtic Rim” of Iberia [81,84]. “The most celticized area of the whole Iberian Peninsula [today] is the north-west” [85]. Together, these observations could explain, in part, the high p.C282Y allele frequencies we observed in the north and northwest of Iberia.

Other investigators posit that *HFE* p.C282Y arose in southern Scandinavia (present-day Denmark, Norway, and Sweden) [86,87] and was spread by the Vikings [14]. The Vikings probably introduced p.C282Y into Iceland and the Faroe Islands, and may have increased p.C282Y allele frequency in other coastal regions [88]. Seafarers from advanced civilizations, Vikings made voyages throughout Europe for exploration, raiding (for plunder, slaves, and ransoms), trade, and settlement during the approximate interval 793–1066 [89,90].

The first Viking raid in Iberia occurred in 844 at Seville about 87 km (54 miles) inland on the Guadalquivir River in the southwest [91]. A three-year Viking campaign (859–861) occurred at Galicia in northwest Iberia [92]. Viking raids continued along the Bay of Biscay and the Atlantic and Mediterranean coasts of Iberia through the early 11th century [91]. The highest *HFE* p.C282Y allele frequencies we tabulated occur in regions adjacent to the coasts, suggestive of past Viking incursions. In contrast, there is no evidence that Vikings settled in Iberia [91].

There was sporadic gene flow from North Africa to Iberia during the Bronze Age, although North African ancestry in Iberia was not widespread until the past 2000 years [93]. By the Roman period (c. 200 BCE–c. 500), there was a major influx of North African ancestry in southern Iberia that continued throughout the Muslim era (Al-Andalus, 711–1492) [93]. This gene flow could account in part for the lower *HFE* p.C282Y allele frequencies we observed in southern Iberia.

The precise age of *HFE* p.C282Y is unknown. Distante et al., considering the estimated age of p.C282Y, migrations, and comparisons of p.C282Y with alleles associated with other inherited disorders, suggested that p.C282Y arose earlier than either the Celtic or Viking period [8]. Other uncertainties in this study include the possibilities that we overlooked one or more published reports of evaluable population/control cohorts, that the same subjects were included in more than one cohort or report, and that there is a non-linear association of p.C282Y allele frequencies with longitude in Spain, Portugal, or Iberia.

A limitation of this study is that we discovered no *HFE* p.C282Y allele frequency data for evaluable population/control cohorts in four autonomous communities of mainland Spain or for Andorra, Pyrénées-Orientales, or Gibraltar [17]. Thus, the decreasing north-to-south gradient of *HFE* p.C282Y allele frequencies described herein may not fully represent that of Iberia. There were no significant effects of geographic area, cohort sample size, or year of cohort recruitment (or year of publication) on *HFE* p.C282Y allele frequencies in Catalonia/Barcelona and Madrid/Madrid population/control cohorts, although there were insufficient data to permit similar evaluations of other cohorts we tabulated. Investigating the factor(s) that contributed to significant deviation of p.C282Y genotypes from HWE proportions in the present population/control cohorts, assessing all of the factors that could account for the variability of p.C282Y allele frequencies in Iberia, and studying regional differences in the prevalence of hemochromatosis associated with p.C282Y homozygosity in Iberia were beyond the scope of this study.

## 5. Conclusions

We conclude that there is a significant decreasing linear north-to-south gradient of *HFE* p.C282Y allele frequencies in Iberia. p.C282Y allele frequencies are highest in regions adjacent to the northwestern coast.

## Figures and Tables

**Table 1:** *HFE* p.C282Y in 25 population/control cohorts in mainland Spain.

Subjects, *n*	Autonomous Community/ Province or City	p.C282Y Homozygotes, *n*	p.C282Y Heterozygotes, *n*	No p.C282Y, *n*	HWE Value of *p* ^a^	p.C282Y Allele Frequency [95% CI]	Author (Year) [Reference]
215	Aragon/Zaragoza	1	14	200	0.1391	0.0372 [0.0230, 0.0596]	Solanos-Barca (2009) [26]
159	Asturias/Oviedo	0	7	152	0.5326	0.0346 [0.0194, 0.0609]	Lauret (2002) [27]
51	Basque Country	0	2	49	0.5050	0.0196 [0.0054, 0.0687]	Baiget (1998) [28]
116	Basque Country/Guipuzcoa	0	12	104	0.6299	0.0517 [0.0298, 0.0882]	de Juan (2001) [29]
281	Basque Country/Barakaldo	0	12	269	0.5565	0.0214 [0.0123, 0.0370]	de Buruaga (2023) [30]
213	Cantabria/Santander	0	19	194	0.6714	0.0446 [0.0287, 0.0686]	Fábrega (2004) [31]
150	Castile-La Mancha/Toledo	0	9	141	0.5581	0.0300 [0.0159, 0.0560]	de Diego (2004) [32]
50	Catalonia	0	3	47	0.5181	0.0300 [0.0103, 0.0845]	Merryweather-Clarke (1997) [12]
348	Catalonia	0	21	327	0.6336	0.0302 [0.0198, 0.0457]	Berez (2005) [33]
108	Catalonia/Barcelona	0	8	100	0.5628	0.0370 [0.0189, 0.0713]	Baiget (1998) [28]
512	Catalonia/Barcelona	1	27	484	0.1955	0.0303 [0.0214, 0.0427]	Sánchez (1998) [34]
5370	Catalonia/Barcelona	8	323	5039	0.2168	0.0316 [0.0285, 0.0351]	Sánchez (2003) [35]
1043	Catalonia/Barcelona	1	60	982	0.4180	0.0292 [0.0228, 0.0373]	Altes (2004) [36]
126	Catalonia/Barcelona	0	8	118	0.5541	0.0278 [0.0135, 0.0562]	Toll (2006) [37]
812	Catalonia/Tarragona	0	51	761	0.1864	0.0308 [0.0234, 0.0404]	Aranda (2007) [38]
179	Extremadura/Badajoz	0	16	163	0.6478	0.0447 [0.0277, 0.0714]	Rodríguez-López (2012) [39]
125	Extremadura/Cáceres	0	5	120	0.5200	0.0200 [0.0086, 0.0460]	Alvarez (2001) [40]
50	Galicia/Santiago de Compostela	1	3	46	0.0505	0.0500 [0.0215, 0.1118]	Soto (2000) [41]
50	Madrid/Madrid	0	2	48	0.5050	0.0200 [0.0055, 0.0700]	de Salamanca (1999) [42]
174	Madrid/Madrid	0	8	166	0.5395	0.0230 [0.0117, 0.0447]	Moreno (1999) [43]
88	Madrid/Madrid	0	3	85	0.5086	0.0300 [0.0159, 0.0560]	Gonzalez-Hevilla (2005) [44]
551	Madrid/Madrid	0	321	230	<0.0001	0.0227 [0.0154, 0.0333]	Ropero-Gradilla (2005) [45]
1000	Madrid/Madrid	0	33	967	0.2964	0.0165 [0.0118, 0.0231]	Ropero (2006) [46]
370	Murcia/Murcia	0	5	365	0.5186	0.0068 [0.0029, 0.0158]	Muro (2007) [47]
94	Valencia/Valencia	1	5	88	0.0561	0.0372 [0.0181, 0.0748]	Monzó (2017) [15]

^a^ Hardy-Weinberg equilibriumvalues of *p* were obtained using exact tests with mid-p adjustments. CI: confidence interval.

**Table 2:** *HFE* p.C282Y in 9 population/control cohorts in mainland Portugal.

Subjects, *n*	Administrative Region ^b^/City	p.C282Y Homozygotes, *n*	p.C282Y Heterozygotes, *n*	No p.C282Y, *n*	HWE Value of *p* ^a^	p.C282Y Allele Frequency [95% CI]	Author (Year) [Reference]
132	Alentejo	0	6	126	0.5282	0.0227 [0.0104, 0.0487]	Cardoso (2001) [16]
116	Algarve	0	2	114	0.5022	0.0086 [0.0024, 0.0308]	Cardoso (2001) [16]
130	Centro	1	10	119	0.1270	0.0462 [0.0266, 0.0790]	Cardoso (2001) [16]
115	Centro/Coimbra	0	5	110	0.5217	0.0217 [0.0093, 0.0498]	Guerreiro (2006) [48]
52	Centro/Viseu	0	0	52	0.5000	0.0000 [0, 0.0356]	Costo-Matos (2013) [49]
133	Lisbon-Tagus Valley	0	8	125	0.5513	0.0301 [0.0153, 0.0582]	Cardoso (2001) [16]
129	Norte	1	13	115	0.2022	0.0581 [0.0355, 0.0936]	Cardoso (2001) [16]
68	Norte/Porto	0	4	62	0.5212	0.0282 [0.0010, 0.0702]	Porto (1998) [50]
146	Norte/Porto	1	8	137	0.0773	0.0342 [0.0187, 0.0618]	Cardoso (2006) [51]

^a^ Hardy-Weinberg equilibrium values of *p* were obtained using exact tests with mid-p adjustments. CI: confidence interval. ^b^ Administrative regions (and their secondary divisions) are: Alentejo (Beja, Évora, and Portalegre); Algarve (Faro); Centro (Aveiro, Castelo Branco, Coimbra, Guarda, Leiria, and Viseu); Lisbon-Tagus Valley (Lisboa, Santarém, and Setúbal); and Norte (Braga, Bragança, Porto, Viana do Castelo, and Vila Real).

**Table 3:** Regional *HFE* p.C282Y allele frequencies in Iberia ^a^.

Region/Country	Allele Frequency
Galicia/Spain	0.0500
Cantabria/Spain	0.0446
Norte/Portugal	0.0419
Aragon/Spain	0.0372
Valencia/Spain	0.0372
Asturias/Spain	0.0346
Catalonia/Spain	0.0312
Lisbon-Tagus Valley/Portugal	0.0301
Castile-La Mancha/Spain	0.0300
Extremadura/Spain	0.0296
Basque Country/Spain	0.0290
Centro/Portugal	0.0286
Alentejo/Portugal	0.0227
Madrid/Spain	0.0200
Algarve/Portugal	0.0086
Murcia/Spain	0.0068

^a^ These regional frequencies were derived from this online dataset [17]. We used single values of *HFE* p.C282Y frequency, as necessary. For two or more p.C282Y frequency reports from the same geographic region [17], we used the quotient of (total number of p.C282Y alleles) by (total number of subjects × 2). See Figure 2.

## Data Availability

The original data analyzed in this study are presented herein or are openly available at MedRXiv (doi: https://doi.org/10.64898/2025.12.19.25342681).

## Abbreviations

The following abbreviations are used in this manuscript:

CI	confidence interval
HWE	Hardy-Weinberg equilibrium
